# 1-Benzyl-3-(1,2-diphenyl­ethen­yl)-1*H*-indole

**DOI:** 10.1107/S1600536810034707

**Published:** 2010-09-04

**Authors:** M. NizamMohideen, G. Bhaskar, P. T. Perumal

**Affiliations:** a Department of Physics, The New College (Autonomous), Chennai 600 014, India; b Organic Chemistry Division, Central Leather Research Institute, Chennai 600 020, India

## Abstract

In the title compound, C_29_H_23_N, the planar [maximum deviation from the least squares plane = 0.056 (1) Å] indole ring makes dihedral angles of 83.4 (4), 69.9 (1) and 59.9 (1)°, with the least-squares planes of three benzene rings. The mol­ecular packing is stabilized by weak inter­molecular C—H⋯π inter­actions.

## Related literature

For applications of heteroarenes, see: Dyker (1999 ▶); Ritleng *et al.* (2002 ▶). For their pharmaceutical properties and for related reactions, see: Sundberg (1996 ▶); Ferrer *et al.* 2007 ▶). For bond-length data, see: Allen *et al.* (1987 ▶).
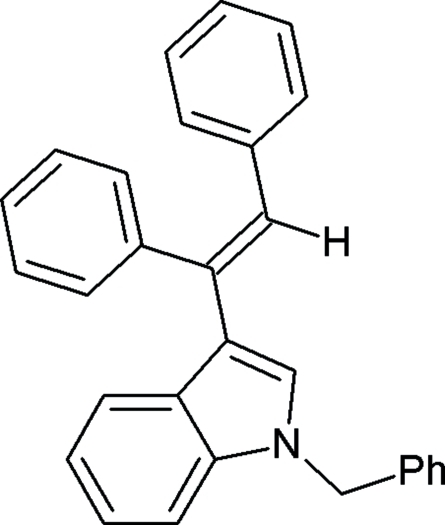


## Experimental

### 

#### Crystal data

C_29_H_23_N
*M* *_r_* = 385.48
Monoclinic, 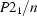

*a* = 9.6513 (7) Å
*b* = 11.1857 (10) Å
*c* = 20.0026 (14) Å
β = 101.636 (4)°
*V* = 2115.0 (3) Å^3^
*Z* = 4
Mo *K*α radiation
μ = 0.07 mm^−1^
*T* = 298 K
0.22 × 0.19 × 0.16 mm

#### Data collection

Bruker Kappa APEXII CCD diffractometer
Absorption correction: multi-scan (*SADABS*; Bruker, 2004 ▶) *T*_min_ = 0.985, *T*_max_ = 0.989
14333 measured reflections
4736 independent reflections
1944 reflections with *I* > 2σ(*I*)
*R*_int_ = 0.057

#### Refinement

*R*[*F* ^2^ > 2σ(*F* ^2^)] = 0.059
*wR*(*F* ^2^) = 0.164
*S* = 0.96
4736 reflections
271 parameters
H-atom parameters constrained
Δρ_max_ = 0.15 e Å^−3^
Δρ_min_ = −0.17 e Å^−3^

### 

Data collection: *APEX2* (Bruker, 2004 ▶); cell refinement: *APEX2* and *SAINT* (Bruker, 2004 ▶); data reduction: *SAINT* and *XPREP* (Bruker, 2004 ▶); program(s) used to solve structure: *SHELXS97* (Sheldrick, 2008 ▶); program(s) used to refine structure: *SHELXL97* (Sheldrick, 2008 ▶); molecular graphics: *ORTEP-3* (Farrugia, 1997 ▶); software used to prepare material for publication: *SHELXL97* and *PLATON* (Spek, 2009 ▶)’.

## Supplementary Material

Crystal structure: contains datablocks global, I. DOI: 10.1107/S1600536810034707/jj2051sup1.cif

Structure factors: contains datablocks I. DOI: 10.1107/S1600536810034707/jj2051Isup2.hkl

Additional supplementary materials:  crystallographic information; 3D view; checkCIF report

## Figures and Tables

**Table 1:** Hydrogen-bond geometry (Å, °) *Cg*1 and *Cg*2 are the centroids of the N1/C1/C2/C3/C8 and C17–C22 rings, respectively.

*D*—H⋯*A*	*D*—H	H⋯*A*	*D*⋯*A*	*D*—H⋯*A*
C9—H9*B*⋯*Cg*1^i^	0.97	2.79	3.619 (3)	144
C28—H28⋯*Cg*2^ii^	0.93	2.92	3.830 (3)	165

Symmetry codes: (i) 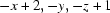; (ii) 
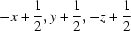
.

**Fig. 1.**
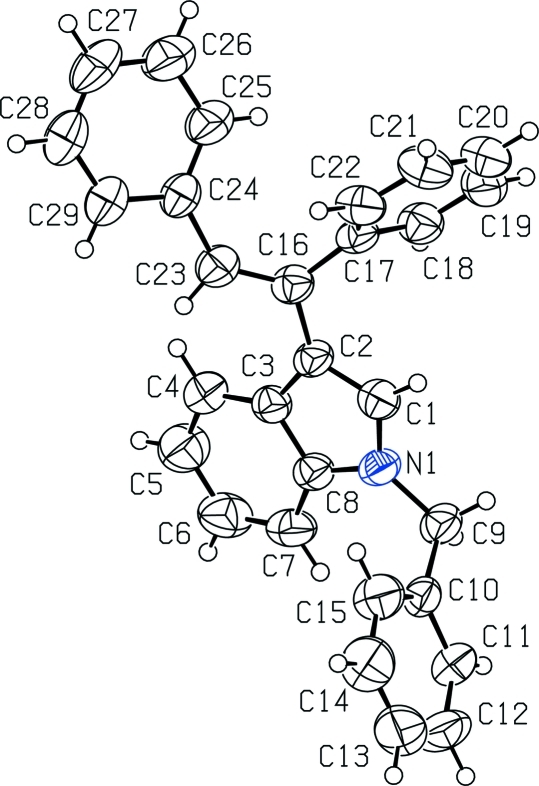
The molecular structure of the title compound, C29H23N, with the atom numbering scheme and 50% probability displacement ellipsoids. H atoms are presented as a small spheres of arbitrary radius.

## supplementary crystallographic information

### Comment

Development of heteroarene functionalization are useful applications such as fluorescent dyes, synthetic analogues of natural products, and pharmaceuticals (Ritleng *et al.*, 2002; Dyker, 1999). The indole ring system exists ubiquitously in natural products, and exhibits important biological and pharmaceutical properties (Sundberg *et al.*, 1996).  A systematic investigation on the gold-catalyzed intra- and intermolecular addition of indoles to alkynes is reported (Ferrer *et al.*, 2007). Against this background the structure of the title compound, C_29_H_23_N, is determined.

In the title compound, the indole ring is planar, the maximum deviation from the least squares plane being 0.056 (1)Å for atom C3 (Fig. 1).  All bond lengths and angles are within normal ranges (Allen *et al.*, 1987). The sum of bond angles around N1 is 350.8 (2)°, indicating *sp*^2^ hybridization.  The dihedral angle formed by the least squares planes of the indole ring and the three benzene rings is 83.4 (4)° (C10—C15), 69.9 (0)° (C17–C21) and 59.9 (0)° (C24–C29), respectively.  The dihedral angle between benzene rings C10—C15 vs C17–C21 and rings C10—C15 vs C24–C29 is 36.7 (6)°.  The molecular packing is stabilized by weak intermolecular C—H···Cg π-ring interactions (Table 1).

### Experimental

A mixture of diphenylacetylene (2.4 mmol),1-benzyl indole (2 mmol), indium tribromide (0.2 mmol) in toluene (4 ml) was stirred at 383° K for 2 hr. After completion of the reaction as indicated by TLC, the reaction mixture was diluted with water and extracted with ethyl acetate.  The combined organic layers were dried over anhydrous Na_2_SO_4_, concentrated *in vacuo* and purified by column chromatography on silica gel (Merck,100–200 mesh) to afford the desired product after crystallization.

### Refinement

All H atoms were positioned geometrically, with C—H = 0.93–0.98 Å and constrained to ride on their parent atoms, with *U*_iso_(H) = *xU*_eq_(C), where *x* = 1.5 for methyl H and *x* = 1.2 for all other H atoms.

### Crystal data

**Table:**

C_29_H_23_N	*F*(000) = 816
*M**_r_* = 385.48	*D*_x_ = 1.211 Mg m^−^^3^
Monoclinic, *P*2_1_/*n*	Mo *K*α radiation, λ = 0.71073 Å
Hall symbol: -P 2yn	Cell parameters from 1467 reflections
*a* = 9.6513 (7) Å	θ = 2.6–20.8°
*b* = 11.1857 (10) Å	µ = 0.07 mm^−^^1^
*c* = 20.0026 (14) Å	*T* = 298 K
β = 101.636 (4)°	Block, colourless
*V* = 2115.0 (3) Å^3^	0.22 × 0.19 × 0.16 mm
*Z* = 4	

### Data collection

**Table:**

Bruker Kappa APEXII CCD diffractometer	4736 independent reflections
Radiation source: fine-focus sealed tube	1944 reflections with *I* > 2σ(*I*)
graphite	*R*_int_ = 0.057
ω and φ scan	θ_max_ = 28.4°, θ_min_ = 2.6°
Absorption correction: multi-scan (*SADABS*; Bruker, 2004)	*h* = −12→11
*T*_min_ = 0.985, *T*_max_ = 0.989	*k* = −13→13
14333 measured reflections	*l* = −25→20

### Refinement

**Table:**

Refinement on *F*^2^	Primary atom site location: structure-invariant direct methods
Least-squares matrix: full	Secondary atom site location: difference Fourier map
*R*[*F*^2^ > 2σ(*F*^2^)] = 0.059	Hydrogen site location: inferred from neighbouring sites
*wR*(*F*^2^) = 0.164	H-atom parameters constrained
*S* = 0.96	*w* = 1/[σ^2^(*F*_o_^2^) + (0.069*P*)^2^] where *P* = (*F*_o_^2^ + 2*F*_c_^2^)/3
4736 reflections	(Δ/σ)_max_ < 0.001
271 parameters	Δρ_max_ = 0.15 e Å^−^^3^
0 restraints	Δρ_min_ = −0.17 e Å^−^^3^

### Special details

**Table:**

Geometry. All e.s.d.'s (except the e.s.d. in the dihedral angle between two l.s. planes) are estimated using the full covariance matrix. The cell e.s.d.'s are taken into account individually in the estimation of e.s.d.'s in distances, angles and torsion angles; correlations between e.s.d.'s in cell parameters are only used when they are defined by crystal symmetry. An approximate (isotropic) treatment of cell e.s.d.'s is used for estimating e.s.d.'s involving l.s. planes.
Refinement. Refinement of *F*^2^ against ALL reflections. The weighted *R*-factor *wR* and goodness of fit *S* are based on *F*^2^, conventional *R*-factors *R* are based on *F*, with *F* set to zero for negative *F*^2^. The threshold expression of *F*^2^ > σ(*F*^2^) is used only for calculating *R*-factors(gt) *etc*. and is not relevant to the choice of reflections for refinement. *R*-factors based on *F*^2^ are statistically about twice as large as those based on *F*, and *R*- factors based on ALL data will be even larger.

### Fractional atomic coordinates and isotropic or equivalent isotropic displacement parameters (Å^2^)

**Table:**

	*x*	*y*	*z*	*U*_iso_*/*U*_eq_	
C1	0.8730 (2)	0.1329 (2)	0.39886 (12)	0.0542 (7)	
H1	0.9018	0.1247	0.3574	0.065*	
C2	0.7345 (2)	0.1302 (2)	0.40656 (12)	0.0491 (6)	
C3	0.7395 (2)	0.1442 (2)	0.47796 (11)	0.0491 (6)	
C4	0.6392 (3)	0.1373 (3)	0.51975 (13)	0.0663 (8)	
H4	0.5440	0.1260	0.5006	0.080*	
C5	0.6829 (3)	0.1474 (3)	0.58865 (14)	0.0832 (9)	
H5	0.6159	0.1437	0.6161	0.100*	
C6	0.8239 (3)	0.1629 (3)	0.61903 (14)	0.0806 (9)	
H6	0.8494	0.1712	0.6662	0.097*	
C7	0.9262 (3)	0.1662 (2)	0.58053 (13)	0.0659 (8)	
H7	1.0213	0.1747	0.6006	0.079*	
C8	0.8821 (2)	0.1563 (2)	0.51037 (12)	0.0503 (6)	
C9	1.1159 (2)	0.1514 (2)	0.47240 (13)	0.0592 (7)	
H9A	1.1458	0.1222	0.4319	0.071*	
H9B	1.1524	0.0967	0.5094	0.071*	
C10	1.1807 (2)	0.2726 (2)	0.49003 (12)	0.0499 (6)	
C11	1.3105 (2)	0.2806 (3)	0.53319 (12)	0.0631 (7)	
H11	1.3531	0.2121	0.5543	0.076*	
C12	1.3780 (3)	0.3894 (4)	0.54542 (16)	0.0867 (10)	
H12	1.4664	0.3936	0.5742	0.104*	
C13	1.3161 (4)	0.4907 (3)	0.51563 (19)	0.0918 (11)	
H13	1.3622	0.5639	0.5240	0.110*	
C14	1.1867 (4)	0.4845 (3)	0.47361 (18)	0.0918 (10)	
H14	1.1435	0.5535	0.4535	0.110*	
C15	1.1194 (3)	0.3754 (3)	0.46090 (15)	0.0755 (8)	
H15	1.0310	0.3718	0.4320	0.091*	
C16	0.6105 (2)	0.1190 (2)	0.35057 (11)	0.0515 (6)	
C17	0.6344 (2)	0.0550 (2)	0.28908 (11)	0.0484 (6)	
C18	0.7060 (2)	−0.0527 (3)	0.29393 (13)	0.0628 (7)	
H18	0.7317	−0.0892	0.3364	0.075*	
C19	0.7404 (3)	−0.1075 (3)	0.23813 (16)	0.0745 (8)	
H19	0.7883	−0.1801	0.2429	0.089*	
C20	0.7036 (3)	−0.0545 (3)	0.17518 (16)	0.0785 (9)	
H20	0.7278	−0.0905	0.1372	0.094*	
C21	0.6326 (3)	0.0498 (3)	0.16852 (13)	0.0719 (8)	
H21	0.6079	0.0855	0.1258	0.086*	
C22	0.5959 (3)	0.1044 (3)	0.22447 (13)	0.0640 (7)	
H22	0.5446	0.1753	0.2187	0.077*	
C23	0.4864 (2)	0.1698 (2)	0.35701 (13)	0.0618 (7)	
H23	0.4919	0.2161	0.3961	0.074*	
C24	0.3455 (2)	0.1647 (3)	0.31340 (12)	0.0562 (7)	
C25	0.2926 (3)	0.0713 (3)	0.27103 (15)	0.0827 (9)	
H25	0.3514	0.0071	0.2668	0.099*	
C26	0.1547 (3)	0.0700 (3)	0.23466 (16)	0.0942 (11)	
H26	0.1217	0.0056	0.2065	0.113*	
C27	0.0660 (3)	0.1649 (4)	0.24024 (17)	0.0891 (10)	
H27	−0.0264	0.1660	0.2152	0.107*	
C28	0.1159 (3)	0.2566 (3)	0.28288 (16)	0.0820 (9)	
H28	0.0565	0.3201	0.2876	0.098*	
C29	0.2521 (3)	0.2569 (3)	0.31884 (13)	0.0702 (8)	
H29	0.2832	0.3207	0.3478	0.084*	
N1	0.96186 (19)	0.14922 (18)	0.46014 (10)	0.0531 (5)	

### Atomic displacement parameters (Å^2^)

**Table:**

	*U*^11^	*U*^22^	*U*^33^	*U*^12^	*U*^13^	*U*^23^
C1	0.0569 (15)	0.0496 (18)	0.0525 (15)	0.0052 (13)	0.0023 (12)	0.0001 (12)
C2	0.0393 (13)	0.0439 (17)	0.0594 (15)	0.0035 (11)	−0.0009 (11)	0.0010 (12)
C3	0.0490 (14)	0.0434 (17)	0.0524 (15)	0.0013 (12)	0.0046 (11)	−0.0014 (12)
C4	0.0513 (15)	0.077 (2)	0.0687 (19)	−0.0006 (14)	0.0067 (14)	−0.0074 (15)
C5	0.077 (2)	0.109 (3)	0.0635 (19)	−0.0081 (19)	0.0151 (16)	−0.0050 (18)
C6	0.088 (2)	0.094 (3)	0.0565 (17)	−0.0118 (19)	0.0061 (17)	−0.0085 (16)
C7	0.0630 (17)	0.062 (2)	0.0637 (18)	−0.0053 (14)	−0.0086 (14)	−0.0053 (14)
C8	0.0466 (14)	0.0391 (17)	0.0623 (16)	−0.0009 (12)	0.0038 (12)	−0.0002 (12)
C9	0.0430 (14)	0.0535 (19)	0.0767 (17)	0.0046 (13)	0.0018 (12)	−0.0007 (14)
C10	0.0411 (14)	0.0489 (19)	0.0594 (15)	0.0035 (13)	0.0096 (12)	0.0018 (13)
C11	0.0459 (15)	0.070 (2)	0.0721 (17)	−0.0049 (14)	0.0088 (13)	0.0033 (15)
C12	0.0548 (18)	0.106 (3)	0.097 (2)	−0.029 (2)	0.0089 (16)	−0.010 (2)
C13	0.092 (3)	0.072 (3)	0.122 (3)	−0.030 (2)	0.047 (2)	−0.017 (2)
C14	0.092 (2)	0.054 (2)	0.130 (3)	−0.001 (2)	0.025 (2)	0.009 (2)
C15	0.0668 (18)	0.057 (2)	0.096 (2)	0.0027 (17)	−0.0005 (16)	0.0042 (18)
C16	0.0485 (14)	0.0480 (17)	0.0537 (15)	0.0026 (13)	0.0001 (11)	0.0043 (13)
C17	0.0416 (13)	0.0454 (17)	0.0522 (15)	−0.0009 (12)	−0.0047 (11)	0.0038 (13)
C18	0.0595 (16)	0.058 (2)	0.0643 (17)	0.0064 (15)	−0.0043 (13)	−0.0011 (15)
C19	0.0684 (18)	0.059 (2)	0.089 (2)	0.0113 (15)	−0.0013 (17)	−0.0144 (19)
C20	0.078 (2)	0.079 (3)	0.077 (2)	−0.0152 (19)	0.0120 (16)	−0.026 (2)
C21	0.092 (2)	0.065 (2)	0.0541 (18)	−0.0093 (18)	0.0034 (15)	0.0006 (16)
C22	0.0698 (17)	0.0533 (19)	0.0616 (18)	−0.0034 (14)	−0.0040 (14)	−0.0019 (14)
C23	0.0535 (16)	0.066 (2)	0.0613 (16)	0.0058 (14)	0.0001 (12)	−0.0008 (13)
C24	0.0477 (14)	0.060 (2)	0.0586 (15)	0.0095 (14)	0.0051 (12)	0.0085 (14)
C25	0.0544 (17)	0.083 (3)	0.102 (2)	0.0073 (16)	−0.0058 (16)	−0.0126 (19)
C26	0.0613 (19)	0.097 (3)	0.113 (3)	0.000 (2)	−0.0087 (18)	−0.013 (2)
C27	0.0467 (17)	0.122 (3)	0.092 (2)	0.012 (2)	−0.0033 (16)	0.021 (2)
C28	0.057 (2)	0.090 (3)	0.097 (2)	0.0196 (18)	0.0126 (17)	0.012 (2)
C29	0.0536 (17)	0.078 (2)	0.0779 (18)	0.0092 (16)	0.0114 (14)	0.0061 (16)
N1	0.0412 (11)	0.0516 (15)	0.0620 (13)	−0.0013 (10)	0.0001 (10)	−0.0011 (10)

### Geometric parameters (Å, °)

**Table:**

C1—N1	1.359 (3)	C14—H14	0.9300
C1—C2	1.377 (3)	C15—H15	0.9300
C1—H1	0.9300	C16—C23	1.355 (3)
C2—C3	1.428 (3)	C16—C17	1.481 (3)
C2—C16	1.470 (3)	C17—C18	1.382 (3)
C3—C4	1.403 (3)	C17—C22	1.386 (3)
C3—C8	1.405 (3)	C18—C19	1.372 (3)
C4—C5	1.362 (3)	C18—H18	0.9300
C4—H4	0.9300	C19—C20	1.372 (4)
C5—C6	1.385 (4)	C19—H19	0.9300
C5—H5	0.9300	C20—C21	1.345 (4)
C6—C7	1.370 (3)	C20—H20	0.9300
C6—H6	0.9300	C21—C22	1.383 (4)
C7—C8	1.386 (3)	C21—H21	0.9300
C7—H7	0.9300	C22—H22	0.9300
C8—N1	1.386 (3)	C23—C24	1.461 (3)
C9—N1	1.457 (3)	C23—H23	0.9300
C9—C10	1.505 (3)	C24—C25	1.377 (4)
C9—H9A	0.9700	C24—C29	1.389 (3)
C9—H9B	0.9700	C25—C26	1.382 (3)
C10—C15	1.369 (4)	C25—H25	0.9300
C10—C11	1.373 (3)	C26—C27	1.383 (4)
C11—C12	1.379 (4)	C26—H26	0.9300
C11—H11	0.9300	C27—C28	1.359 (4)
C12—C13	1.361 (4)	C27—H27	0.9300
C12—H12	0.9300	C28—C29	1.365 (3)
C13—C14	1.359 (4)	C28—H28	0.9300
C13—H13	0.9300	C29—H29	0.9300
C14—C15	1.381 (4)		
			
N1—C1—C2	110.6 (2)	C14—C15—H15	119.4
N1—C1—H1	124.7	C23—C16—C2	119.4 (2)
C2—C1—H1	124.7	C23—C16—C17	124.8 (2)
C1—C2—C3	105.7 (2)	C2—C16—C17	115.79 (19)
C1—C2—C16	125.2 (2)	C18—C17—C22	116.7 (2)
C3—C2—C16	129.0 (2)	C18—C17—C16	121.5 (2)
C4—C3—C8	117.4 (2)	C22—C17—C16	121.6 (2)
C4—C3—C2	134.5 (2)	C19—C18—C17	122.1 (3)
C8—C3—C2	107.75 (19)	C19—C18—H18	118.9
C5—C4—C3	119.2 (2)	C17—C18—H18	118.9
C5—C4—H4	120.4	C18—C19—C20	119.6 (3)
C3—C4—H4	120.4	C18—C19—H19	120.2
C4—C5—C6	122.0 (3)	C20—C19—H19	120.2
C4—C5—H5	119.0	C21—C20—C19	119.8 (3)
C6—C5—H5	119.0	C21—C20—H20	120.1
C7—C6—C5	120.9 (3)	C19—C20—H20	120.1
C7—C6—H6	119.6	C20—C21—C22	120.8 (3)
C5—C6—H6	119.6	C20—C21—H21	119.6
C6—C7—C8	117.3 (2)	C22—C21—H21	119.6
C6—C7—H7	121.4	C21—C22—C17	120.9 (3)
C8—C7—H7	121.4	C21—C22—H22	119.5
N1—C8—C7	129.5 (2)	C17—C22—H22	119.5
N1—C8—C3	107.19 (19)	C16—C23—C24	131.4 (2)
C7—C8—C3	123.2 (2)	C16—C23—H23	114.3
N1—C9—C10	114.6 (2)	C24—C23—H23	114.3
N1—C9—H9A	108.6	C25—C24—C29	116.5 (2)
C10—C9—H9A	108.6	C25—C24—C23	125.6 (2)
N1—C9—H9B	108.6	C29—C24—C23	117.7 (3)
C10—C9—H9B	108.6	C24—C25—C26	122.0 (3)
H9A—C9—H9B	107.6	C24—C25—H25	119.0
C15—C10—C11	118.3 (3)	C26—C25—H25	119.0
C15—C10—C9	122.3 (2)	C25—C26—C27	119.7 (3)
C11—C10—C9	119.3 (2)	C25—C26—H26	120.1
C10—C11—C12	120.5 (3)	C27—C26—H26	120.1
C10—C11—H11	119.8	C28—C27—C26	118.9 (3)
C12—C11—H11	119.8	C28—C27—H27	120.6
C13—C12—C11	120.5 (3)	C26—C27—H27	120.6
C13—C12—H12	119.7	C27—C28—C29	121.0 (3)
C11—C12—H12	119.7	C27—C28—H28	119.5
C14—C13—C12	119.7 (3)	C29—C28—H28	119.5
C14—C13—H13	120.2	C28—C29—C24	121.9 (3)
C12—C13—H13	120.2	C28—C29—H29	119.1
C13—C14—C15	119.9 (3)	C24—C29—H29	119.1
C13—C14—H14	120.0	C1—N1—C8	108.66 (18)
C15—C14—H14	120.0	C1—N1—C9	126.2 (2)
C10—C15—C14	121.1 (3)	C8—N1—C9	125.02 (19)
C10—C15—H15	119.4		
			
N1—C1—C2—C3	−0.9 (3)	C2—C16—C17—C18	46.9 (3)
N1—C1—C2—C16	177.3 (2)	C23—C16—C17—C22	49.6 (4)
C1—C2—C3—C4	−172.3 (3)	C2—C16—C17—C22	−128.0 (2)
C16—C2—C3—C4	9.6 (5)	C22—C17—C18—C19	1.3 (4)
C1—C2—C3—C8	0.7 (3)	C16—C17—C18—C19	−173.8 (2)
C16—C2—C3—C8	−177.4 (2)	C17—C18—C19—C20	0.3 (4)
C8—C3—C4—C5	2.6 (4)	C18—C19—C20—C21	−0.9 (4)
C2—C3—C4—C5	175.2 (3)	C19—C20—C21—C22	0.0 (4)
C3—C4—C5—C6	−0.8 (5)	C20—C21—C22—C17	1.7 (4)
C4—C5—C6—C7	−1.4 (5)	C18—C17—C22—C21	−2.3 (4)
C5—C6—C7—C8	1.5 (4)	C16—C17—C22—C21	172.9 (2)
C6—C7—C8—N1	−175.4 (2)	C2—C16—C23—C24	−174.0 (2)
C6—C7—C8—C3	0.5 (4)	C17—C16—C23—C24	8.5 (4)
C4—C3—C8—N1	174.1 (2)	C16—C23—C24—C25	28.5 (4)
C2—C3—C8—N1	−0.3 (3)	C16—C23—C24—C29	−157.1 (3)
C4—C3—C8—C7	−2.6 (4)	C29—C24—C25—C26	1.3 (4)
C2—C3—C8—C7	−177.0 (2)	C23—C24—C25—C26	175.7 (3)
N1—C9—C10—C15	−36.5 (3)	C24—C25—C26—C27	0.2 (5)
N1—C9—C10—C11	148.1 (2)	C25—C26—C27—C28	−1.5 (5)
C15—C10—C11—C12	−1.4 (4)	C26—C27—C28—C29	1.2 (5)
C9—C10—C11—C12	174.2 (2)	C27—C28—C29—C24	0.3 (4)
C10—C11—C12—C13	0.9 (4)	C25—C24—C29—C28	−1.6 (4)
C11—C12—C13—C14	0.1 (5)	C23—C24—C29—C28	−176.5 (2)
C12—C13—C14—C15	−0.6 (5)	C2—C1—N1—C8	0.7 (3)
C11—C10—C15—C14	0.9 (4)	C2—C1—N1—C9	177.1 (2)
C9—C10—C15—C14	−174.6 (3)	C7—C8—N1—C1	176.2 (2)
C13—C14—C15—C10	0.1 (5)	C3—C8—N1—C1	−0.3 (3)
C1—C2—C16—C23	−149.8 (3)	C7—C8—N1—C9	−0.2 (4)
C3—C2—C16—C23	27.9 (4)	C3—C8—N1—C9	−176.7 (2)
C1—C2—C16—C17	27.9 (4)	C10—C9—N1—C1	109.4 (3)
C3—C2—C16—C17	−154.3 (2)	C10—C9—N1—C8	−74.8 (3)
C23—C16—C17—C18	−135.6 (3)		

### Hydrogen-bond geometry (Å, °)

**Table:**

Cg1 and Cg2 are the centroids of the N1/C1/C2/C3/C8 and C17–C22 rings, respectively.

**Table:**

*D*—H···*A*	*D*—H	H···*A*	*D*···*A*	*D*—H···*A*
C9—H9B···Cg1^i^	0.97	2.79	3.619 (3)	144
C28—H28···Cg2^ii^	0.93	2.92	3.830 (3)	165

Symmetry codes: (i) −*x*+2, −*y*, −*z*+1; (ii) −*x*+1/2, *y*+1/2, −*z*+1/2.
